# The Predictive Value of Serum Erythropoietin Levels Measured at Diagnosis in Patients With Myelodysplastic Syndromes

**DOI:** 10.1002/jha2.70366

**Published:** 2026-07-29

**Authors:** Dominic Culligan, Adele Taylor, Alexandra Smith, Catherine Cargo, Howard Oster, Argiris Symeonidis, Reinhard Stauder, Saskia Langemeijer, Luca Malcovati, Pierre Fenaux, Jaroslav Čermák, Eva Hellström‐Lindberg, Juliet Mills, Ioannis Kotsianidis, Maud D'Aveni, Corine van Marrewijk, Marlijn Hoeks, Theo de Witte, David Bowen, Moshe Mittelman

**Affiliations:** ^1^ Department of Haematology Aberdeen Royal Infirmary Aberdeen UK; ^2^ Epidemiology and Cancer Statistics Group, Department of Health Sciences University of York York UK; ^3^ Haematological Malignancy Diagnostic Service, Leeds Cancer Centre St James's University Hospital & Leeds Teaching Hospitals NHS Trust Leeds UK; ^4^ Department of Medicine and The Department of Hematology Tel Aviv Sourasky (Ichilov) Medical Center and Medical Faculty Tel Aviv University Tel Aviv Israel; ^5^ Department of Medicine Division of Hematology University of Patras Medical School Olympion Hospital & Rehabilitation Center Patras Greece; ^6^ Department of Internal Medicine V (Haematology and Oncology) Innsbruck Medical University Innsbruck Austria; ^7^ Department of Public Health Health Services Research and Health Technology Assessment UMIT TIROL – University for Health Sciences and Technology Hall in Tirol Austria; ^8^ Department of Hematology Radboud University Medical Center Nijmegen The Netherlands; ^9^ Department of Hematology Oncology Fondazione IRCCS Policlinico San Matteo University of Pavia Pavia Italy; ^10^ Service d'Hématologie, Hôpital Saint‐Louis Assistance Publique des Hôpitaux de Paris (AP‐HP) and Université Paris 7 Paris France; ^11^ Department of Clinical Hematology Institute of Hematology & Blood Transfusion Praha Czech Republic; ^12^ Department of Medicine Division Hematology Center For Hematology and Regenerative Medicine Karolinska Institutet Stockholm Sweden; ^13^ Department of Haematology Worcestershire Acute Hospitals NHS Trust and University Hospitals Birmingham NHS Foundation Trust Birmingham UK; ^14^ Department of Hematology University Hospital of Alexandroupolis Democritus University of Thrace Medical School Alexandroupolis Greece; ^15^ Hematology Department CHRU‐Nancy Université De Lorraine Nancy France; ^16^ Department of Tumor Immunology‐Nijmegen Center for Molecular Life Sciences Radboud University Medical Center Nijmegen The Netherlands; ^17^ St. James's Institute of Oncology Leeds Teaching Hospitals Leeds UK

**Keywords:** ESA response, leukemia‐free survival, myelodysplastic syndromes, overall survival, prognosis, serum erythropoietin levels, transfusion free survival

## Abstract

**Objectives**: To study the predictive and prognostic values of serum erythropoietin levels (sEPO) measured at diagnosis in lower‐risk myelodysplastic syndromes.

**Methods**: We analyzed clinical associations and prognosis with sEPO in 672/1610 patients with at least one year of follow‐up data in the prospective EUMDS study.

**Results**: sEPO levels increase with WHO subtypes associated with erythroid hypoplasia and poorer risk IPSS‐R scores. sEPO is an independent predictor of transfusion free survival and levels < 200 IU/L predict response to ESA. sEPO is not an independent predictor of overall survival.

**Conclusions**: sEPO predicts transfusion free survival and response to ESA.

**Trial Registration**: The EUMDS Registry (ClinicalTrials.gov: Identifier NCT00600860).

## 1 Introduction

Anaemic patients with myelodysplastic syndromes (MDS) tend to have raised serum erythropoietin (sEPO) levels, varying from normal to > 10,000 IU/L [1, 2]. Erythropoiesis stimulating agents (ESA) are the commonest first line treatment for MDS‐related anaemia [3] and sEPO has served as a reliable predictor of response to ESA [3]. However, the wider predictive value of sEPO levels has only been reported in small studies [4, 5]. Given the survival benefit with ESA response and transfusion independence [6], we analysed the associations and predictive value of sEPO levels, measured within three months of initial diagnosis, in a cohort of 1610 patients from the European MDS Registry. This is a real world, prospective, multi‐center, non‐interventional study of newly diagnosed (< 100 days) patients with predominantly lower‐risk MDS (IPSS‐R very low, low, and intermediate). Prospective data and clinical management are recorded at six monthly visits as previously detailed [6]. sEPO levels are not mandated for recruitment, but measured at the enrolling clinicians’ discretion and according to local policy.

## 2 Patients and Methods

Eligible IPSS lower‐risk MDS patients from 16 European countries and Israel had a minimum of three data points from study entry (Visit 1), month six (Visit 2) and month 12 (Visit 3) and were without elevated baseline serum creatinine (> 106.1 umol/L [1.2 mg/dL]). We investigated whether baseline sEPO levels showed a relationship with demographic and clinical factors including MDS subtype (WHO 2016 classification) and International Prognostic Scoring System‐Revised (IPSS‐R). The relationship between having sEPO measured and the likelihood of receiving red blood cell (RBC) transfusions or ESA therapy at each visit were studied. Using standard descriptive methods baseline sEPO levels were summarised by the median and interquartile range and compared between groups using the Kruskal–Wallis test. Owing to high skewness of the distribution, we used log base 10 transformation on baseline sEPO for further analyses. sEPO as a predictor of outcomes was analysed using standard time‐to‐event methods. These included RBC‐transfusion free survival (TFS) defined as the time lapsed from diagnosis to first RBC transfusion and hematological improvement erythroid (HI‐E) with ESA therapy defined as an increase in Hb of at least 1.5 g/dL (modified IWG 2006 [7]) from the visit before starting ESA compared to the visit after. TFS and ESA response are partly linked but are amongst the most important outcomes for patients keen to avoid transfusions and maintain quality of life [6]. We also studied leukemia free survival (LFS) defined as time from diagnosis to progression to AML or death, progression free survival (PFS) defined as time from diagnosis to progression to higher‐risk MDS or death and overall survival (OS), defined as time from diagnosis to death from any cause. Patients without an event were censored at their last known visit date. Cox proportional hazard models described univariate analyses and multivariate analyses adjusted for baseline haemoglobin, platelets, absolute neutrophil count, bone marrow blasts, ring sideroblasts and IPSS‐R cytogenetic risk group. Time on ESA was measured from the date of starting ESA to the date of stopping and censored at last visit for ongoing treatment. All analyses were undertaken in Stata 18.0 (StataCorp, College Station, TX).

## 3 Results

In total, 1610 patients were eligible (Table 1); 672 patients (41.7%) had a baseline sEPO level measured. There was no difference by age, but females were more likely to have their sEPO level measured. Differences were seen by diagnosis and IPSS‐R; patients with MDS with ring sideroblasts (MDS‐RS) and MDS with isolated del(5q) were more likely to be tested than those with high/very high IPSS‐R scores, suggesting that the main incentive for EPO measurement is its predictive value for response to ESA therapy. The proportion of patients having baseline sEPO recorded varied between countries, ranging from 6.0% in Israel to 88.3% in the Czech Republic. This in part reflects the wide variation in reimbursed access to ESA during the study period [6]. In terms of therapeutic interventions, at registration 26.8% of patients had received an RBC transfusion and 17.1% ESA therapy. Patients with a recorded sEPO level at diagnosis were more likely to receive RBC transfusions compared with patients who did not: 30.8% versus 24.0% at Visit 1 (*χ* ^2^ = 9.27, *p* = 0.002), 31.1% versus 25.9% at Visit 2 (*χ* ^2^ = 5.23, *p* = 0.022) and 32.0% versus 23.8% at Visit 3 (*χ* ^2^ = 13.50, *p* < 0.001; Table 1 and Table S1). Patients who had sEPO recorded were also more likely to receive ESA therapy between Visits 1 and 3: 38.2% versus 30.6%, (*χ* ^2^ = 10.23, *p* < 0.001) for Visit 2; 39.6% versus 31.0%, (*χ* ^2^ = 12.84, *p* < 0.001) for Visit 3 (Table S2).

**TABLE 1:** Baseline patient characteristics by serum EPO status.

	All patients *N* (%)	Patients with sEPO measured at diagnosis *N* (%)
**Total**	1610 (100)	672 (41.7)
**Sex**
Males	940 (58.4)	372 (55.4)
Females	670 (41.6)	300 (44.6)
**Age at diagnosis (years)** Median (range)	72 (18–93)	72 (21–91)
**Diagnosis WHO 2016**		
MDS‐SLD	242 (15.0)	81 (12.1)
MDS‐RS	375 (23.3)	199 (29.6)
MDS‐MLD	648 (40.2)	268 (39.9)
MDS‐EB	178 (11.1)	60 (8.9)
MDS with isolated del(5q)	103 (6.4)	52 (7.7)
MDS/MPN	2 (0.1)	0 (0.0)
MDS‐unclassifiable	61 (3.8)	11 (1.6)
Other	1 (0.1)	1 (0.1)
**International Prognostic Scoring System—Revised**		
Very Low	424 (26.3)	180 (26.8)
Low	740 (46.0)	325 (48.4)
Intermediate	265 (16.5)	103 (15.3)
High/Very High	44 (2.7)	24 (3.6)
Unknown	137 (8.5)	40 (6.0)
**Red cell transfusion status at Visit 1**		
Yes	432 (26.8)	207 (30.8)
No	1178 (73.2)	465 (69.2)
**ESA therapy status at Visit 1**		
Yes	276 (17.1)	119 (17.7)
No	1334 (82.9)	553 (82.3)
**Country**		
Austria	111 (6.9)	31 (4.6)
Croatia	11 (0.7)	6 (0.9)
Czech Republic	102 (6.3)	85 (12.6)
Denmark	48 (3.0)	37 (5.5)
France	298 (18.5)	116 (17.3)
Germany	39 (2.4)	21 (3.1)
Greece	198 (12.3)	54 (8.0)
Israel	167 (10.4)	10 (1.5)
Italy	68 (4.2)	38 (5.7)
The Netherlands	84 (5.2)	24 (3.6)
Poland	30 (1.9)	11 (1.6)
Portugal	27 (1.7)	22 (3.3)
Romania	26 (1.6)	21 (3.1)
Serbia	23 (1.4)	17 (2.5)
Spain	68 (4.2)	45 (6.7)
Sweden	77 (4.8)	66 (9.8)
UK	233 (14.5)	68 (10.1)

Abbreviations: IPSS‐R, Revised International Prognostic Scoring System; MDS‐EB, MDS with excess blasts; MDS‐MLD, MDS with multilineage dysplasia; MDS‐RS, MDS with ring sideroblasts; MDS‐SLD, MDS with single lineage dysplasia.

**FIGURE 1:**
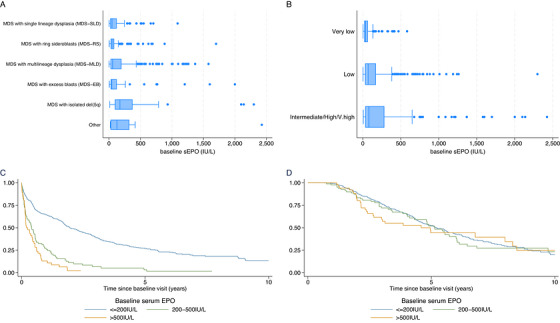
Distribution of sEPO levels (Median; 25th–75th centile) according to (A) WHO 2016 categories and (B) IPSS‐R prognostic groups. (C) transfusion free survival and (D) overall survival according to baseline (Visit 1) sEPO levels.

The distribution of sEPO levels, expressed as a continuous variable by baseline WHO 2016 diagnoses and IPSS‐R categories, is depicted in Figure 1a,b and in Table S3. Higher baseline sEPO levels were seen in patients with MDS with multilineage dysplasia as well as in MDS with isolated del(5q), which demonstrated the highest levels of baseline sEPO in keeping with well‐recognized red cell hypoplasia (*χ* ^2^ = 43.1, *p* = 0.0001; Figure 1a and Table S3). MDS‐RS demonstrated one of the lowest sEPO levels, corresponding with the well‐known increased percentage of bone marrow RBC precursors observed in this patient group and to recently described accumulation of immature erythroblasts in *SF3B1* mutated MDS [8]. Similarly, sEPO levels exhibited a significantly increased trend on moving through the IPSS‐R prognostic groups from very low to intermediate/high/very high, *χ* ^2^ = 53.60, *p* = 0.0001 (Figure 1b and Table S3). Reflecting that the Registry started in 2008, inclusion was based on Low or Intermediate‐1 IPSS risk; we subsequently re‐assigned patients to the IPPS‐R and only 44 (6%) of patients were in the high/very high category, in keeping with predominantly lower‐risk MDS patients. When comparing IPSS‐R cytogenetic risk groups, the lowest sEPO levels occurred in the very good cytogenetic risk group.

Baseline sEPO levels were strongly associated with TFS on univariate analysis (Figure 1c hazard ratio [HR] 3.26; 95% confidence intervals (95% CI): 2.72–3.89, *p* < 0.001). sEPO remained an independent predictor of TFS (HR 1.94; CI 1.58–2.39) when adjusted for factors detailed above (Table S4). Response could be assessed for 353 patients who had a follow‐up visit with recorded Hb after commencing ESA; sEPO <200 IU/L was associated with a higher rate of HI‐E with ESA than 200–500 IU/L and > 500 IU/L (*χ* ^2^ = 14.83, *p* = 0.001; Table S5). Median time on ESA therapy was 1.44 years (1.23–1.59); for sEPO ≤ 200 IU/L 1.96 years (1.44–2.24), sEPO 200–499 IU/L 0.56 years (0.36–0.90) and sEPO > 500 IU/L 1.04 years (0.54–1.20). Whilst univariate analysis showed that higher baseline sEPO levels were associated with poorer LFS (HR 1.57; 1.32–1.87), PFS (HR 1.32; 1.09–1.59) and OS (HR 1.26; 1.05–1.52), none of these maintained the same level of significance when adjusted as detailed above; 1.07 (0.87–1.33), 0.93 (0.75–1.17), 0.88 (0.70–1.10), respectively (Figure 1d and Figure S1a,b).

## 4 Discussion

Despite over thirty years of measuring sEPO, the prognostic significance of sEPO beyond predicting response to ESA remains unclear [9]. Interestingly, the rate of baseline sEPO assessments varied significantly by country, from 6% to 88.3%, in part reflecting access and re‐imbursement arrangements for ESA therapy, or EPO measurement, and consistent with highly variable ESA use within the registry [6]. This influences the generalisability of our results across health systems. Current first line interventions in LR‐MDS are predominantly ESA therapy and RBC transfusions. We demonstrated that having a baseline sEPO level recorded correlated with increased likelihood of receiving RBC transfusions or with starting ESA within a year of registration. This suggests physicians measure sEPO prior to therapeutic interventions, most likely assessing the benefit of ESA therapy by way of the Nordic [3] or alternative [9] scores. As such, there is indication bias in the non‐mandated decision to measure sEPO. These predictive scores were devised when patients were often diagnosed later with higher sEPO levels. However, newer predictors of response to ESAs, incorporating molecular gene mutation data (IPSS‐M) and specific sex‐associated somatic gene mutation data, still support the value of sEPO when included in the model [10]. As such, sEPO remains relevant despite increasing use of the IPSS‐M.

sEPO is in part regulated by the rate of EPO utilisation by erythropoiesis [11]. We demonstrated sEPO levels significantly increased with higher‐risk MDS diagnostic subgroups and IPSS‐R prognostic groups as erythropoiesis likely decreases. However, the highest sEPO was in the del(5q) favorable subgroup, in keeping with well documented erythroid hypoplasia. Other hypoplastic MDS subtypes have demonstrated high sEPO levels [12]. We observed the lowest levels in patients with MDS‐RS and MDS with single lineage dysplasia (SLD), likely reflecting an expanded erythroblast mass in MDS‐RS [8]. This may explain, in part, why low sEPO levels are associated with higher responses to ESA as suggested in the recent COMMANDS trial [13] and confirmed in this study. As such, we believe sEPO < 200 U/L should become the standard definition of ESA eligibility in future trials. COMMANDS has led to licensing of luspatercept in first line. Predicting response to luspatercept is complex. Real‐world data suggests poor response at low sEPO (< 80 IU/l) [14], perhaps reflecting its action on later stage, EPO independent, erythropoiesis. One drawback is that methodologies for measuring EPO vary and sensitivity differs between ELISA and IRMA with high inter‐laboratory variation [15].

Higher sEPO predicted a poorer TFS on univariate and multivariate analyses adjusted for baseline hemoglobin, platelets, absolute neutrophil count, bone marrow blasts, ring sideroblasts and IPSS‐R cytogenetic risk group. A ten‐fold increase in sEPO increased the odds of transfusion at 12 months on study by 5.5 (95% CI: 3.9–7.9). Earlier transfusion likely results from higher baseline sEPO levels dissuading physicians, who have options, from starting ESAs or leading to earlier ESA failure. While we did not show an OS benefit with low sEPO levels, our propensity matching study of ESA therapy demonstrated that achieving transfusion independence was associated with improved OS [6].

In conclusion, sEPO increases with WHO subtypes associated with erythroid hypoplasia and poorer risk IPSS‐R scores. sEPO helps inform patients of the timescale for requiring red cell transfusions, though not OS. Patients with sEPO levels < 200 U/L are more likely to respond to ESA and have a better TFS.

## Author Contributions

**Dominic Culligan**, **David Bowen** and **Moshe Mittelman** were involved in study design, data collection, analysis and interpretation of the data and writing and editing the first draft. **Adele Taylor** and **Alex Smith** were involved in study design, extracting, statistically analysing and interpreting the data and correcting the first draft. **Luca Malcovati**, **Corine van Marrewijk**, and **Theo de Witte** were involved in funding acquisition, supervision, and contributed to study design, interpreting the data and critical review and correction of the first draft. Catherine Cargo, Howard Oster, Argiris Symeonidis, Reinhard Stauder, Saskia Langemeijer, Pierre Fenaux, Jaroslav Čermák, Eva Hellström‐Lindberg, Juliet Mills, Ioannis Kotsianidis, Maud D'Aveni, and Marlijn Hoeks contributed to data collection. All authors critically reviewed the manuscript and approved its final revision.

## Funding

The EUMDS Registry is supported by an educational grant from Novartis Pharmacy B.V. Oncology Europe, Bristol‐Myers Squibb/Celgene International, Amgen Limited, Janssen Pharmaceutica, Takeda Pharmaceuticals International, Lumanity RWE Limited and Gilead Sciences Inc.

## Ethics Statement

The EUMDS Registry was approved by each institution's Ethics Committee in accordance with national legislation.

## Consent

All participants in the EUMDS Registry Study provided informed consent.

## Conflicts of Interest

**CC** reports honoraria from Novartis and Astellas, travel support from Novartis; **ASy** reports honoraria from Abbvie, Astra‐Zeneca, BMS, Glaxo, Janssen, Novartis, Pfizer, Sanofi; and participation in advisory/DSM board for Abbvie, Astra‐Zeneca, BMS, Glaxo, Janssen, Novartis, Sanofi, Sobi; **RS** reports honoraria from BMS for the MDS Right Horizon 2020 Project, financial support for meetings from Celgene/BMS, Lilly, Teva, AbbVie, and AHOP; advisory board participation for BMS and Otsuka; and is Servier President of Verein Senioren Krebshilfe. **IK** reports research funding from BMS; lecture honoraria, meeting/travel support, and participation in advisory/DSM board for BMS and Janssen; **MdA** reports consulting fees from BMS; meeting/travel support from Sanofi; **CvM,** project manager of the EUMDS Registry, is funded from the EUMDS (educational grants from Novartis Pharmacy B.V. Oncology Europe, BMS/Celgene International, Amgen Limited, Janssen Pharmaceutica, Takeda Pharmaceuticals International, Lumanity RWE Limited and Gilead Sciences Inc); **MM** reports consulting fees from BioCenvergence, TALENT Group and Dr. Reddy's; honoraria for lectures from Dr. Reddy's and FibroGen; and meeting/travel support from FibroGen; **DJC, AT, ASm, HO, SL, LM, PF, JC, EHL, JM, MH, TdW** and **DB** have no relevant conflicts of interest to declare.

## Supporting information




**Supporting File**: jha270366‐sup‐0001‐SupMat.docx

## Data Availability

The data sets generated during this study are based on data from the European MDS Registry (www.EUMDS.org). The data are not publicly available due to privacy or ethical restrictions. Access to data that support the findings of this study can be obtained from the EUMDS project management office upon reasonable request. A fee might be required.
