# Supplementary material for: The Predictive Value of Serum Erythropoietin Levels Measured at Diagnosis in Patients With Myelodysplastic Syndromes
Source: EJHaem. 2026 Jul 29;7(4):e70366. doi: 10.1002/jha2.70366 (PMC13418852; doi:10.1002/jha2.70366)
Supplement: Supplementary file 1 — Supporting File: jha270366‐sup‐0001‐SupMat.docx [file JHA2-7-e70366-s001.docx]

**Supplementary Tables and Figures**

**Supplementary Table 1: Baseline Serum EPO levels and treatment with Red Blood Cell transfusions by visit**

|  |  | | |  |
| --- | --- | --- | --- | --- |
|  | **Transfused Patients**  **N (%)** | **sEPO measured at baseline** | |  |
|  |  | **Yes**  **N (%)** | **No**  **N (%)** | **P value** |
| Total |  | 672 | 938 |  |
|  |  |  |  |  |
| Visit |  |  |  |  |
| V1 (baseline) | 432 (26.8) | 207 (30.8) | 235 (24.0) | 0.002 |
|  |  |  |  |  |
| V2 | 452 (28.1) | 209 (31.1) | 243 (25.9) | 0.022 |
|  |  |  |  |  |
| V3 | 438 (27.2) | 215 (32.0) | 223 (23.8) | <0.001 |
|  |  |  |  |  |

**Supplementary Table 2: Serum EPO levels and treatment with ESA at each visit time point**

|  |  | | |  |
| --- | --- | --- | --- | --- |
|  | **Patients received ESA**  **N (%)** | **sEPO measured at baseline** | |  |
|  |  | **Yes**  **N (%)** | **No**  **N (%)** | **P value** |
| Total |  | 672 | 938 |  |
|  |  |  |  |  |
| Visit |  |  |  |  |
| V1 (baseline) | 276 (17.1) | 119 (17.7) | 157 (16.7) | NS |
|  |  |  |  |  |
| V2 | 544 (33.8) | 257 (38.2) | 287 (30.6) | <0.001 |
|  |  |  |  |  |
| V3 | 557 (34.6) | 266 (39.6) | 291 (31.0) | <0.001 |
|  |  |  |  |  |

**Supplementary Table 3: Serum EPO by baseline characteristics**

|  |  | Serum EPO IU/L | |
| --- | --- | --- | --- |
|  | N | Mean (sd) | Median (25th - 75th) |
| Total | 672 | 162 (292) | 58 (28 - 150) |
|  |  |  |  |
| **Diagnosis as per WHO 2016:** |  |  |  |
| MDS with single lineage dysplasia (MDS-SLD) | 81 | 125 (190) | 49 (21 - 123) |
| MDS with ring sideroblasts (MDS-RS) | 199 | 95 (173) | 49 (28 - 83) |
| MDS with multilineage dysplasia (MDS-MLD) | 268 | 173 (268) | 60 (26 - 198) |
| MDS with excess blasts (MDS-EB) | 60 | 178 (370) | 46 (29 - 124) |
| MDS with isolated 5q- | 52 | 349 (503) | 175 (95 - 374) |
| Other | 12 | 345 (670) | 127 (26 - 322) |
|  |  |  |  |
| **International Prognostic Scoring System – Revised:** |  |  |  |
| Very low | 180 | 66 (92) | 35 (20 - 66) |
| Low | 325 | 160 (245) | 71 (31 - 173) |
| Intermediate/High/Very High | 167 | 267 (444) | 80 (33 - 284) |
|  |  |  |  |
| **IPSS-R Cytogenetic risk group:** |  |  |  |
| Very good | 47 | 101 (172) | 35 (19 - 82) |
| Good | 491 | 164 (293) | 60 (29 - 154) |
| Very poor/Poor/Intermediate | 123 | 182 (330) | 60 (29 - 178) |
|  |  |  |  |

IPSS-R = International Prognostic Scoring System - Revised

**Supplementary Table 4: Hazard Ratios for transfusion free survival estimated by Cox’s proportional regression**

|  | Adjusted Hazard ratio (95% CI)^1^ | p-value |
| --- | --- | --- |
| Log transformed serum EPO (IU/L) | 1.943 (1.580 - 2.388) | <0.0001 |
| Haemoglobin | 0.660 (0.614 - 0.709) | <0.0001 |
| Platelet count | 0.999 (0.999 - 1.000) | 0.019 |
| Neutrophil count | 0.974 (0.921 - 1.030) | 0.363 |
| Bone marrow blasts (%) | 1.009 (0.963 - 1.058) | 0.693 |
|  |  |  |
| **IPSS-R cytogenetic risk group** |  |  |
| Very poor/Poor/Intermediate | 1 (reference) | . |
| Good | 0.762 (0.599 - 0.968) | 0.026 |
| Very good | 0.916 (0.600 - 1.398) | 0.685 |
| **Ring sideroblasts** |  |  |
| No | 1 (reference) | . |
| Yes (diagnosis of MDS-RS) | 0.899 (0.721 - 1.121) | 0.346 |

^1^ adjusted for variables included in the table

**Supplementary Table 5: Response to ESA therapy according to baseline (Visit 1) sEPO level being: < 200 IU/L, 200-500 IU/L or > 500 IU/L**

|  | **sEPO measured at baseline (iU/l)** | | |  |
| --- | --- | --- | --- | --- |
|  | <=200 | 200-499 | >500 | Total |
|  |  |  |  |  |
| Total | 297 (84.1) | 38 (10.8) | 18 (5.1) | 353 (100.0) |
|  |  |  |  |  |
| No response to ESA | 201 (82.4) | 35 (14.3) | 8 (3.3) | 244 (100.0) |
| Response to ESA | 96 (88.1) | 3 (2.8) | 10 (9.2) | 109 (100.0) |

χ^2^ = 14.83 P = 0.0014

**A**

**
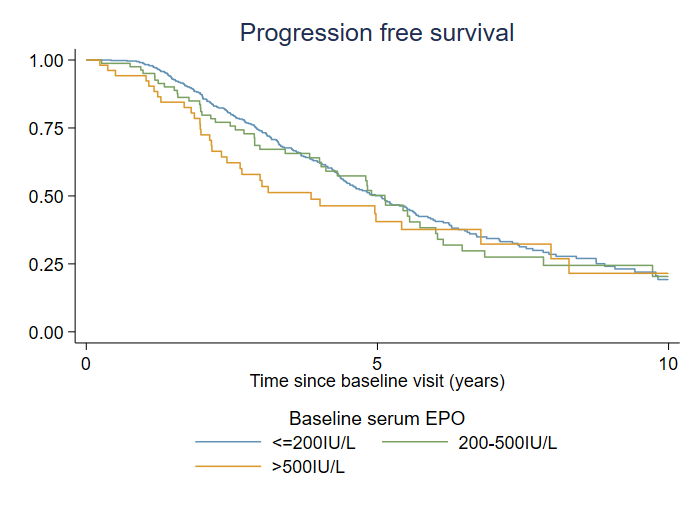
**

**B**

**
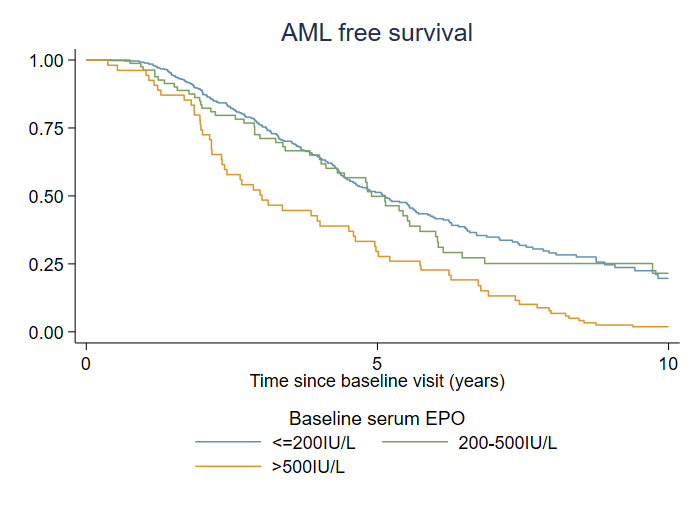
**

**Supplementary Figure 1: [A]** Progression free survival and **[B]** AML free survival according to baseline (visit 1) sEPO levels.

**Supplementary Table 6:**

| **Country** | **Participating centres** (City, Name organization, *[local investigator(s)]*) |
| --- | --- |
| **Austria** | Innsbruck, Medical University of Innsbruck *[R. Stauder* */* ***V. Petzer*** */* ***D. Wolf****]* |
|  | Wels, Klinikum Wels-Grieskirchen *[S. Heibl]* |
|  | Lienz, Bezirkskrankenhaus *[A. Walder]* |
|  | Vienna, Hanusch Krankenhaus *[M. Pfeilstöcker / A. Schoenmetzler-Makrai]* |
| **Croatia** | Zagreb, Clinical Hospital Merkur *[****I. Mandac Smoljanovic****]* |
| **Czech Republic** | Prague, General University Hospital, Institute of Hematology and Blood Transfusion *[****J. Cermak*** */ D. Mikulenková]* |
|  | Brno, University Hospital Brno-Bohunice *[L. Cervinek]* |
|  | Prague, Motol University Hospital *[J. Segethová]* |
|  | Prague, General University Hospital, 1st Clinic of Internal Medicine *[A. Jonasova]* |
|  | Hradec Kralove, Charles University Faculty Hospital *[P. Belohlavkova]* |
|  | Olomouc, University Hospital *[R. Machova]* |
| **Denmark** | Aarhus, University Hospital *[*M.S. Holm*]* |
|  | Odense, Odense University Hospital *[H. Vestergaard]* |
|  | Copenhagen, University Hospital: Rigshospitalet *[L. Kjeldsen / K. Grønbæk /* ***J. Werner Hansen****]* |
|  | Herlev Ringvej, Herlev Hospital *[I.H. Dufva]* |
|  | Aalborg, University Hospital *[P.D. Jensen]* |
| **France** | Bobigny, Hospital Avicenne *[P. Fenaux]* |
|  | Paris, Hôpital St. Louis *[****P. Fenaux*** */ R. Itzykson / L. Adès]* |
|  | Nancy, CHU Nancy: Hospital Brabois (Vandoeuvre Les Nancy) *[M. D’Aveni]* |
|  | Toulouse, CHU Toulouse: Hospital Purpan, Toulouse *[T. Comont]* |
|  | Avignon, Centre Hospital *[B. Slama]* |
|  | Perpignan, Centre Hospital Maréchal Joffre *[L. Sanhes]* |
|  | Lyon, Hospital Edouard Herriot *[E. Wattel]* |
|  | Chalon sur Saone, Centre Hospital William Morey *[D. Klepping / B. Salles]* |
|  | Paris, Hospital Cochin *[L. Willems]* |
|  | Tours, CHRU de Tours *[E. Gyan]* |
|  | Limoges, CHU Limoges Hospital Dupuytren *[M. Gourin]* |
|  | Strasbourg, CHU Hospital Hautepierre de Strasbourg *[S. Amé]* |
|  | Caen, Centre Hospital Universitaire Clemenceau *[S. Cheze]* |
|  | Boulogne sur Mer, Centre Hospital Boulogne-sur-Mer *[B. Choufi]* |
|  | Le Kremlin Bicêtre, Hospital Bicêtre *[G. Tertian]* |
|  | Lille - St Vincent, Hospital St Vincent de Paul *[L. Pascal]* |
|  | Nice, CHU de Nice: Hospital l'Archet *[T. Cluzeau]* |
|  | Clermont-Ferrand, Centre Hospital Universitaire *[B. de Renzis]* |
|  | Grenoble, CHU Albert Michallon *[S. Park]* |
|  | Pontoise, Centre Hospital René Dubos Pontoise *[R. Benramdane]* |
|  | Rouen, CHU de Rouen: Hospital Charles-Nicolle *[A. Stamatoullas]* |
|  | Ivry sur Seine, Hôpital Charles-Foix Ap-Hp *[V. Siguret]* |
| **Germany** | Düsseldorf, Heinrich-Heine University Hospital *[****U. Germing****]* |
|  | Dresden, University Hospital Carl Gustav Carus *[U. Platzbecker]* |
|  | Duisburg, HELIOS: St. Johannes Hospital in Hamborn *[C. Badrakan]* |
|  | Ulm, University Hospital Ulm *[R. Schlenk]* |
| **Greece** | Patras, General University Hospital of Patras *[****A. Symeonidis*** */ A. Kourakli]* |
|  | Alexandroupolis, Democritus University of Thrace *[I. Kotsianidis / C. Tsatalas]* |
|  | Athens, General Hospital Laikon - Propaedeutic Medicine, University of Athens Medical School *[P. Panagiotidis]* |
|  | Athens, Patission Prefectural General Hospital: Halkida *[Z. Kartasis]* |
|  | Athens, Pammakaristos Hospital *[A. Kostourou]* |
|  | Patras, St. Andreas General Hospital *[P. Zikos]* |
|  | Chania, General Hospital of Chania *[K. Palla]* |
|  | Ioannina, University Hospital of Ioannina *[V. Briasoulis / E. Hatzimichael]* |
|  | Piraeus, Metaxa Hospital *[M. Kotsopoulou / K. Megalakaki]* |
|  | Athens, General Hospital G. Gennimatas *[A. Galanopoulos / E. Michali]* |
|  | Athens, General Hospital Laikon - Internal Medicine, University of Athens Medical School *[N. Viniou]* |
|  | Serres, General Hospital of Serres *[M. Protopapa]* |
|  | Pilea Chortiatis, General Hospital of Thessaloniki George Papanikolaou *[A. Anagnostopoulos]* |
|  | Athens, General Hospital Attikon, University of Athens Medical School *[V. Pappa]* |
|  | Thessaloniki, Hippokration - General Hospital of Thessaloniki *[E. Vlachaki]* |
|  | Athens, Hellenic 251 Air Force General Hospital *[E. Terpos]* |
|  | Athens, General Hospital Sotiria, University of Athens Medical School  *[A. Katsigiannis / P. Roussou]* |
|  | Athens, St. Savvas Oncology Hospital of Athens *[A. Pouli]* |
|  | Thessaloniki, Theageneio General Hospital *[P. Konstantinidou]* |
| **Israel** | Tel Aviv, Tel Aviv Sourasky (Ichilov) Medical Centre *[****M. Mittelman****]* |
|  | Rehovot, Kaplan Medical Center *[K. Filanovsky]* |
|  | Kfar Saba, Meir Medical Center *[I. Hellman / M. Ellis]* |
|  | Nahariya, The Western Galilee Hospital *[G. Stemer]* |
|  | Be'er Ya'akov, Asaf-Harofe Medical Center *[U. Gotwin / O. Cohen / M. Koren]* |
|  | Haifa, Rambam Medical Centre *[N. Glaubach]* |
|  | Ramat Gan, Sheba Medical Center *[M. Drorit ]* |
|  | Beersheba, Soroka Medical Center *[E. Levy / U. Greenbaum]* |
|  | Petah Tikva, Rabin Medical Center *[ O. Wolaj]* |
|  | Netanya, Laniado Hospital *[S. Bolvik]* |
|  | Ashkelon, Barzilai Medical Center *[A. Nemetz]* |
|  | Afula, HaEmek Medical Center *[D. Oquasha]* |
|  | Haifa, Carmel Medical Center *[M. Price]* |
|  | Jerusalem, Hadassah Medical Center *[N. Goldshmidt / S. Elias / R. Saban]* |
|  | Haifa, Bnai Zion Medical Center *[S. Gino-Moor]* |
|  | Tiberias, Baruch Padeh Medical Center Poriya *[S. Yeganeh / N. Benyamini]* |
|  | Holon, Wolfson Medical Center *[N. Sarid]* |
|  | Safed, Ziv Medical Center *[N. Dali]* |
| **Italy** | Pavia, University of Pavia Medical School, IRCCS San Matteo Hospital Foundation *[****L. Malcovati****]* |
|  | Rome, University Cattolica del Sacro Cuore, Policlinico Gemelli *[L. Fianchi]* |
| **Netherlands** | Den Bosch, Jeroen Bosch Hospital *[A. Herbers / H. Pruijt]* |
|  | Nijmegen, Radboudumc *[****S. Langemeijer*** */* ***M. Hoeks****]* |
|  | Uden, Bernhoven Hospital *[C. Lensen]* |
|  | Ede, Gelderse Vallei Hospital *[G. Velders]* |
|  | Arnhem, Rijnstate Hospital *[M. Cuijpers]* |
|  | Amsterdam, VU University Medical Center *[A. vd Loosdrecht]* |
|  | Doetinchem, Slingeland Hospital *[N. Aboosy]* |
|  | Helmond, Elkerliek Hospital *[E. Jacobs]* |
|  | Veldhoven, Maxima Medical Center *[P. Kuijper]* |
| **Poland** | Warsaw, Medical University of Warsaw *[****K. Madry****]* |
| **Portugal** | Lisbon, Centro Hospitalar de Lisboa *[M. Câmara]* |
|  | Lisbon, Hospital Da Luz *[****A. Almeida****]* |
| **Romania** | Bucharest, Fundeni Clinical Institute *[****A. Tatic****]* |
|  | Bucharest, Coltea Clinical Hospital *[O. Stanca]* |
|  | Brasov, Districtual Hospital *[G. Vulcan]* |
| **Serbia** | Novi Sad, Clinical Center of Vojvodina *[****A. Savic****]* |
| **Spain** | Oviedo, Hospital Universitario Central de Asturias *[T. Bernal]* |
|  | Valencia, Hospital Universitario La Fe *[****G. Sanz****]* |
|  | Valencia, Hospital Clinico Universitario de Valencia *[D. Tormo]* |
|  | Barcelona, Hospital del Mar *[C. Pedro]* |
|  | Lleida, Instituto de Investigación Biomédica *[V. Betés]* |
|  | Barcelona, Hospital Universitari Germans Trias i Pujol *[B. Xicoy]* |
|  | Murcia, Hospital Universitario Meseguer *[M. Lozano / M. Martínez]* |
|  | Valencia, Hospital Dr. Peset *[R. Andreu Lapiedra]* |
|  | Salamaca, Hospital Universitario de Salamanca *[M. Diez Campelo]* |
|  | Cádiz, Hospital Universitario Puerta del Mar *[J. Muñoz]* |
| **Sweden** | Stockholm, Karolinska University hospital *[****E. Hellström-Lindberg****]* |
|  | Göteborg, Sahlgrenska University Hospital *[H. Garelius]* |
|  | Stockholm, Södersjukhuset *[M. Grövdal]* |
|  | Umeå, Umeå regional hospital *[F. Lorenz]* |
|  | Linköping, University Hospital Linköping *[P. Antunovic / A. Jönsson]* |
|  | Halmstad, Teaching Hospital of Halmstad *[C. Karlsson]* |
|  | Uppsala, Uppsala University *[E. Ejerblad]* |
|  | Eskilstuna, Mälarsjukhuset *[E. Hesse Sundin]* |
|  | Lund, Lund University Hospital *[L. Nilsson]* |
|  | Örebro, Örebro University Hospital *[P.Kozlowski]* |
| **United** | Aberdeen, Aberdeen Royal Infirmary *[D. Culligan]* |
| **Kingdom** | Worcester, Worcestershire Acute Hospitals NHS Trust *[S. Hebballi]* |
|  | Leeds, Leeds Teaching Hospitals NHS Trust *[****C. Cargo*** */ M. Karakantza]* |
|  | Harrogate, Harrogate District Hospital *[C. Hall]* |
|  | Blackpool, Blackpool Victoria Hospital *[S. Kolade / P. Cahalin]* |
|  | Steeton, Airedale NHS Trust *[E. Nga]* |
|  | York, York Hospital *[L. Bond]* |
|  | Truro, Royal Cornwall Hospital *[D. Creagh]* |
|  | Bradford, Bradford Royal Infirmary *[S. Ackroyd]* |
|  | Bournemouth, Royal Bournemouth Hospital *[S. Killick]* |
|  | Northampton, Northampton General Hospital *[J. Parker / A. Bowen]* |
|  | Wakefield, Mid Yorkshire Hospitals *[J. Ashcroft]* |
|  | Cambridge, Addenbrooke's Hospital *[C. Wong / A. Warren]* |
|  | Glasgow, Western Infirmary *[M. Drummond]* |
|  | Huddersfield, Huddersfield Royal Infirmary *[K. Rothwell]* |
|  | Nottingham, City Hospital *[R. Radia / E. Das-Gupta]* |
|  | Hull, Hull and East Yorkshire Hospitals NHS Trust *[S. Green / S. Ali]* |
|  | Newcastle upon Tyne, Royal Victoria Infirmary *[G. Jones]* |
|  | Oxford, John Radcliffe Hospitals NHS Trust *[P. Vyas]* |

The National Principal Investigators are highlighted in bold, and the Steering Committee Members are underlined.
